# Prognostic tools or clinical predictions: Which are better in palliative care?

**DOI:** 10.1371/journal.pone.0249763

**Published:** 2021-04-28

**Authors:** P. Stone, V. Vickerstaff, A. Kalpakidou, C. Todd, J. Griffiths, V. Keeley, K. Spencer, P. Buckle, D. Finlay, R. Z. Omar

**Affiliations:** 1 Division of Psychiatry, Marie Curie Palliative Care Research Department, University College London (UCL), London, United Kingdom; 2 Faculty of Biology, Medicine and Health, School of Health Sciences, The University of Manchester, Manchester, United Kingdom; 3 Manchester Academic Health Science Centre, Manchester, United Kingdom; 4 Manchester University NHS Foundation Trust, Manchester, United Kingdom; 5 Palliative Medicine Department, University Hospitals of Derby and Burton NHS Foundation Trust, Derby, United Kingdom; 6 Department of Statistical Science, University College London (UCL), London, United Kingdom; University of Technology Sydney, AUSTRALIA

## Abstract

**Purpose:**

The Palliative Prognostic (PaP) score; Palliative Prognostic Index (PPI); Feliu Prognostic Nomogram (FPN) and Palliative Performance Scale (PPS) have all been proposed as prognostic tools for palliative cancer care. However, clinical judgement remains the principal way by which palliative care professionals determine prognoses and it is important that the performance of prognostic tools is compared against clinical predictions of survival (CPS).

**Methods:**

This was a multi-centre, cohort validation study of prognostic tools. Study participants were adults with advanced cancer receiving palliative care, with or without capacity to consent. Key prognostic data were collected at baseline, shortly after referral to palliative care services. CPS were obtained independently from a doctor and a nurse.

**Results:**

Prognostic data were collected on 1833 participants. All prognostic tools showed acceptable discrimination and calibration, but none showed superiority to CPS. Both PaP and CPS were equally able to accurately categorise patients according to their risk of dying within 30 days. There was no difference in performance between CPS and FPN at stratifying patients according to their risk of dying at 15, 30 or 60 days. PPI was significantly (p<0.001) worse than CPS at predicting which patients would survive for 3 or 6 weeks. PPS and CPS were both able to discriminate palliative care patients into multiple iso-prognostic groups.

**Conclusions:**

Although four commonly used prognostic algorithms for palliative care generally showed good discrimination and calibration, none of them demonstrated superiority to CPS. Prognostic tools which are less accurate than CPS are of no clinical use. However, prognostic tools which perform similarly to CPS may have other advantages to recommend them for use in clinical practice (e.g. being more objective, more reproducible, acting as a second opinion or as an educational tool). Future studies should therefore assess the impact of prognostic tools on clinical practice and decision-making.

## Introduction

Prognostic information is essential for informing decision-making at the end of life. Patients’ understanding about their prognoses is often inaccurate and over-optimistic [1–5]. Patients expect their physicians to provide them with honest accurate and realistic estimates of survival [6,7]. However, although clinicians’ estimates are frequently better than patients’ own predictions [1,5], they still tend to be inaccurate [8,9]. For this reason physicians are encouraged to supplement their clinical intuition with validated prognostic algorithms [10,11]. A number of such prognostic tools have been developed for use in patients with advanced cancer [12,13]. The performance of relatively few of these tools has been compared against clinicians’ own predictions of survival [14–18].

The Prognosis in Palliative Care Study (PiPS) was a multi-centre prospective study to develop and validate a prognostic tool for cancer palliative care [19]. Prognostic models were developed to predict 14-day and 56-day survival in either patients for whom blood results were not (PiPS-A) or were (PiPS-B) available. PiPS-A and PiPS-B risk categories (predicted survival of “days”, “weeks” or “years”) were found to be as accurate as an agreed multi-professional (doctor and nurse) estimate of survival [19]. The PiPS2 study [20] was a prospective multi-centre validation of various prognostic tools including the PiPS-A and PiPS-B 14-day and 56-day models and the corresponding risk categories. The primary analysis demonstrated that all of the models (PiPS-A14, PiPS-A56, PiPS-B14 and PiPS-B56) had excellent discrimination and were well-calibrated. However, only the PiPS-B risk categories were found to be as accurate as an agreed multi-professional survival estimate [21].

In addition to validating PiPS, the PiPS2 study also evaluated four other prognostic models: Palliative Prognostic Index (PPI) [22], Palliative Performance Scale (PPS) [23], Palliative Prognostic (PaP) [24] score, and Feliu Prognostic Nomogram (FPN) [25]. PPI and PPS can both be calculated without the need for a blood test (like PiPS-A). PaP and FPN, both require blood test results (like PiPS-B). This report describes the evaluation of these prognostic scores in a cohort of advanced incurable cancer patients and compares their performance against clinicians’ own predictions of survival.

## Methods

This was a multi-centre, prospective, cohort, validation study of prognostic models. The protocol has been published and registered (ISRCTN 13688211) [20]. The study received approval from Yorkshire and Humber-Leeds East Research Ethics Committee (16/YH/0132).

### Population

The study involved patients, with or without capacity to consent to participate, from 27 UK palliative care services. Patients were recruited from community and hospital palliative care teams, and from inpatient palliative care units. Capacity to participate was assessed by the Principal Investigator (or delegate) at each site [26]. Eligible patients with capacity were approached by a member of the clinical team, handed a Patient Information Sheet, and invited to provide written informed consent to participate. For patients without capacity a personal consultee was sought for advice. For patients with no personal consultee, the advice of a nominated consultee was sought.

### Inclusion criteria

- Incurable cancer
- 18 years or over
- Recent referral to palliative care services
- For patients with capacity, the ability to read and understand the Patient Information Sheet. For patients without capacity, the approval of a personal or professional consultee was required.

#### Exclusion criterion

Treatment with curative intent.

### Data collection

Predictor data were obtained from a review of the medical notes, from discussion with clinical staff and/or directly from patients. The data required for the calculation of each of the prognostic scores is shown in Table 1. Additional data were collected for the calculation of PiPS prognostic scores, which have been presented elsewhere [21].

**Table 1:** Variables required for the calculation of each prognostic score.

Variable type	Variable name	PaP	FPN	PPI	PPS
**Assessments by clinician**	Clinician Prediction of survival	x			
	Eastern Co-operative Oncology Group		x		
	Karnofsky Performance Scale	x			
	Palliative Performance Scale (PPS)			x	x
	Time to terminal disease		x		
**Blood tests**	Albumin		x		
	Lactate Dehydrogenase		x		
	Lymphocyte count	x	x		
	White blood count	x			
**Clinical signs and symptoms**	Anorexia	x			
	Delirium			x	
	Dyspnoea at rest	x		x	
	Oedema			x	
	Oral intake			x	

#### Palliative Prognostic (PaP) score

PaP scores classify patients into three risk groups based on a 30-day survival probabilities of less than 30%; between 30–70%; and more than 70%. Higher scores predict shorter survival. PaP scores are generated by applying a “weighted‟ score to each of six variables (clinician prediction of survival, Karnofsky performance status, anorexia, dyspnoea, total white blood count and lymphocyte percentage).

#### Feliu Prognostic Nomogram (FPN)

FPN uses five variables (Eastern Cooperative Oncology Group [ECOG] performance status [27], serum albumin, Lactate Dehydrogenase, lymphocyte counts and time from initial diagnosis to diagnosis of terminal disease [TTD]) to predict the probability of survival at 15, 30 and 60 days.

#### Palliative Performance Scale (PPS)

PPS is a measure of functional status specifically developed for use in palliative care. PPS is scored by HCPs and consists of 10 categories. A score of 10% represents the poorest functional status (totally bed bound, unable to do any activity, mouth care only and drowsy or in a coma). A score of 100% represents the best functional status (full ambulation, normal activity and work with no evidence of disease, able to take care of themselves, normal intake of food and full conscious level).

#### Palliative Prognostic Index (PPI)

PPI is calculated using five clinical variables (from the Palliative Performance Scale [PPS], oral intake, the presence or absence of dyspnoea, oedema and delirium). The model stratifies into three groups; survival shorter than three weeks (score >6), shorter than six weeks (score >4), or more than six weeks (score < = 4).

#### Clinical prediction of survival

The attending doctor and nurse estimated survival of study participants independently. To maximize the available data for comparisons with prognostic tools a hierarchical approach was taken to produce a clinical prediction of survival (CPS). Doctors’ predictions were used when available and nurses’ predictions were used when no doctors’ predictions were provided. Clinicians were asked to provide their prognostic estimates using a number of different formats in order to facilitate comparison with outputs of prognostic scores. Clinicians were asked: to provide approximate estimates of length of survival—“days” (0–13 days); “weeks” (14–55 days); “months+” (56+ days); to provide more specific estimates of survival to the nearest week (from <1 week to >12 weeks); and to estimate the probability of survival at specific time points (1 day; 3 days; 7 days; 15 days; 30 days and 60 days).

Doctors and nurses in the PiPS2 study were asked to provide some data about themselves, including age, gender, specialty, years working as a doctor, years of experience in palliative medicine and their professional grade. However, individual clinicians were not identified.

#### Survival

Dates of death were obtained from NHS Digital (**https://digital.nhs.uk/**) at least three months after the last participant had been recruited.

### Outcomes

Primary outcomes were survival of patients (from the date of enrolment), predictions of survival made by clinicians and prognostic scores. The gold standard for survival analyses was the absolute survival of patients, and all of the tools studied (and the performance of the clinicians themselves) were first evaluated against this gold standard. We then evaluated the performance of prognostic tools against clinical predictions of survival.

### Methods of analysis and sample size calculation

#### Sample size

The primary aim of this study was to validate the PiPS-B risk categories [21] and the sample size of 1778 participants was predicated on this. The rationale for the sample size has been published elsewhere [21]. It has been recommended that validation data for risk models should have at least 100 events [28]. There is no guidance on sample size calculation for multi-centre prognostic validation studies. To be conservative, we inflated the number of events to validate the prognostic models to 150. Assuming an event rate of 17.8%, based on the original study, we estimated that we would require 843 patients to validate the PiPS-B risk categories. Therefore, the proposed sample size for the primary outcome was considered to be adequate to also validate the other prognostic models presented here (PPI, PPS, PaP and FPN).

#### Statistical analyses

Prognostic tools were evaluated in the form in which they were originally presented and for which they were intended to be used. Discrimination refers to the ability to distinguish between patients with different risks of survival. When possible, the discriminatory ability of the risk tools was assessed using the C-statistic for binary outcomes and Harrell’s C-index for survival outcomes. Discrimination was also assessed graphically using Kaplan-Meir survival curves. Calibration is the agreement between observed and predicted outcomes. Calibration was assessed using the calibration slope based on a Cox model for the Feliu Prognostic Nomogram (FPN) [29] as this is the only risk tool which presents specific predicted probabilities for individual patients. Calibration was also assessed by comparing the predicted and observed proportions of patients surviving to specific time-points for those models which made such predictions.

*PaP*. Since the PaP score stratifies patients into one of three prognostic groups, clinicians were similarly asked to stratify patients into the same three groups (<30%, 30–70% and >70% probability of surviving 30 days). The PaP does not make a prediction about whether or not patients will or will not survive for 30 days, and so the accuracy of individual predictions could not be assessed. Model performance of PaP was assessed by plotting the Kaplan-Meier survival curve for each of the three risk groups. We determined the median survival of each prognostic group to see whether it fell in the expected range. We expected patients with a <30% probability of survival to have a median survival of fewer than 30 days, we expected patients with a >70% probability of survival to have a median survival of greater than 30 days and we expected patients with a 30–70% probability of surviving 30 days to have a median survival of approximately 30-days. We also compared the observed proportion of study participants, in each risk group, who died within 30 days with the proportion predicted by PaP and the proportion predicted by clinicians respectively. PaP provides probability windows rather than specific probabilities for survival. In order to calculate a c-statistic for the model we used the midpoints of the windows (ie. 85%, 50% and 15%).

*FPN*. The nomogram can be used to predict the probability that patients will survive for 15, 30 or 60 days. Because the FPN provides a probabilistic rather than a temporal prediction of survival, it was not possible to make a straightforward comparison between the accuracy of the FPN prediction and the accuracy of clinician predictions. However, clinicians were similarly asked to estimate the probability of patients surviving for 15, 30 and 60 days and the observed proportion of the patient population who survived for these times could be compared against the model and the clinicians’ predictions respectively. The C-index for the FPN was also calculated.

*PPI*. In contrast to the PaP and the FPN the PPI produces a specific prediction about whether patients will live for <3 weeks; 3–6 weeks; or >6 weeks. Predictions were considered to be correct if the patient died/survived for the predicted length of time. Since clinicians were also asked to specifically make predictions about whether patients would survive to these time points it was possible to make a direct comparison between this models and the clinicians. McNemar’s test was used to compare the proportion of overall patient deaths predicted correctly by PPI with the corresponding proportion predicted correctly by clinicians. A C-statistic was calculated separately for the performance of the 3-week and the 6-week models. The performance of the model was further evaluated by plotting the Kaplan-Meier survival curve for each of the three risk groups identified by PPI.

*PPS*. The PPS was not specifically designed as a prognostic tool. We assessed performance of the PPS as a prognostic indicator by plotting the Kaplan-Meier survival curve for each of the ten PPS levels. We compared this with the ability of clinicians to categorise patients into 10 iso-prognostic groups according to their probability of surviving 30-days. Finally, in order to calculate a C-statistic for the PPS, we compared its performance against previously published probabilities of 30-day survival according to PPS categories [30].

## Results

A total of 1833 participants (1610 with; 223 without capacity) were enrolled in the study. The median survival of the participants from enrolment was 45 days (IQ Range 16 to 140).

Participant characteristics are shown in Table 2.

**Table 2:** Participant characteristics.

Variable	
Age (years); mean (SD); n = 1832^*^	70·2 (11·9)
Gender; n (%); n = 1832^*^	
Male	938 (51·2)
Female	894 (48·8)
**Location; n (%)**	
Inpatient Palliative Care Unit	1241 (67·7)
Community Palliative Care Team	468 (25·5)
Hospital Palliative Care Team	124 (6·8)
Site of Primary tumour**; n (%)	
Lung	362 (19·8)
Upper GI tract	337 (18·4)
Head and neck	280 (15·3)
Prostate	160 (8·7)
Breast	146 (8·0)
Gynaecological	133 (7·3)
Other	123 (6·7)
Urological (bladder, testes, renal)	112 (6·1)
Lower GI tract	81 (4·4)
Haematological	70 (3·8)
Unknown	45 (2·5)
Neurological	38 (2·1)
Rare tumour	27 (1·5)
**Site of metastatic diseases; n (%)**	
Bone	555 (30·3)
Liver	538 (29·4)
Nodal	516 (28·2)
Lung	477 (26·0)
Other	353 (19·3)
None	279 (15·2)
Brain	134 (7·3)
Pleural effusion	98 (5·4)
Ascites	95 (5·2)
Adrenal	79 (4·3)
Unknown	60 (3·3)
Skin	36 (2·0)
Renal	20 (1·1)
**Currently receiving tumour therapy; yes n (%)**	391 (21·3)
**If yes, type of therapy:**	
Chemotherapy	190 (48·6)
Radiotherapy	118 (30·2)
Hormone therapy	76 (19·4)
Other tumour directed therapy (e.g. immunotherapy)	42 (10·7)
**Capacity to consent; n (%)**	1610 (87·8)
**Time between diagnosis and date became incurable; n = 1821**	
Mean (SD); months	13·2 (32·8)
Median (IQ Range); months	0 (0, 12)
**Abbreviated Mental Test Score (AMTS); n = 1826**	
Less than 4	208 (11·4)
Greater or equal 4	1618 (88·6)
**Presence or absence of key symptoms**	
Anorexia; yes; n = 1830	968 (52·9)
Dysphagia; yes; n = 1830	554 (30·3)
Dyspnoea; yes; n = 1831	652 (35·6)
Fatigue; yes; n = 1831	1617 (88·3)
Lost weight; yes; n = 1831	1194 (65·2)
**Clinical assessments**	
Ascites; n = 1830	245 (13·4)
Presence of peripheral oedema; n = 1831	685 (37·4)
Pulse rate; beats/min; mean (SD); n = 1817	82·2 (14·7)
Presence of delirium; n = 1830	66 (3·6)
If Yes, is it considered to be caused by a single medication	2 (3·0)
Oral intake; n = 1830	
Normal	587 (32·1)
Moderately reduced	666 (36·4)
Severely reduced	577 (31·5)
**Eastern Co-operative Oncology Group score (ECOG) Performance status; n = 1831**	
Grade 0	15 (0·8)
Grade 1	202 (11·0)
Grade 2	520 (28·4)
Grade 3	822 (44·9)
Grade 4	272 (14·9)
**Global health status (overall health); n (%); n = 1823**	
1 (Very poor)	144 (7·9)
2	414 (22·7)
3	680 (37·3)
4	348 (19·1)
5	180 (9·9)
6	49 (2·7)
7 (Excellent)	8 (0·4)
**Karnofsky Performance Scale (KPS); n (%); n = 1830**	
10	63 (3·4)
20	108 (5·9)
30	136 (7·4)
40	229 (12·5)
50	465 (25·4)
60	404 (22·1)
70	276 (15·1)
80	114 (6·2)
90	33 (1·8)
100	2 (0·1)
**Full blood count**	**Mean (SD)**
White blood count (x10^9^/L); n = 1602	11·3 (11·2)
Lymphocyte count (x10^9^/L); n = 1596	1·2 (2·0)
Neutrophil count (x10^9^/L); n = 1600	8·8 (6·2)
Platelets (x10^9^/L); n = 1601	312·9 (147·6)
**Biochemistry**	
Urea (mmol/L); n = 1601	8·0 (6·4)
Albumin (g/L); n = 1600	30·1 (7·0)
Alkaline phosphatase (U/L); n = 1587	231·7 (319·9)
Alanine transaminase (U/L); n = 1581	33·3 (71·7)
C reactive protein (mg/L); n = 1565	68·6 (73·5)
Lactate Dehydrogenase (mmol/L); n = 1467	505·4 (446·0)

Notes for Table 2

* One participant preferred not to say.

** 73 participants had more than one primary tumour.

### Characteristics of clinician participants

Although individual clinicians were not identified, we assumed that participants working in the same institution who were of the same gender and age and had the same professional grade, years qualified and years working in palliative care were the same individual. On that basis, we concluded that most doctors were palliative care specialists (86%; 360/420). Doctors had a mean of 12.8 (SD 9.7) years’ post-qualification experience and 6.2 years’ (SD 7.1) experience of palliative care. Nearly all of the nurse participants worked in palliative care (98%; 765/768). They had a mean of 19.4 (SD 11.8) years of post-qualification experience and 8.5 years (SD 7.6) working in palliative care.

### Palliative Prognostic Score (PaP)

**Fig 1:**
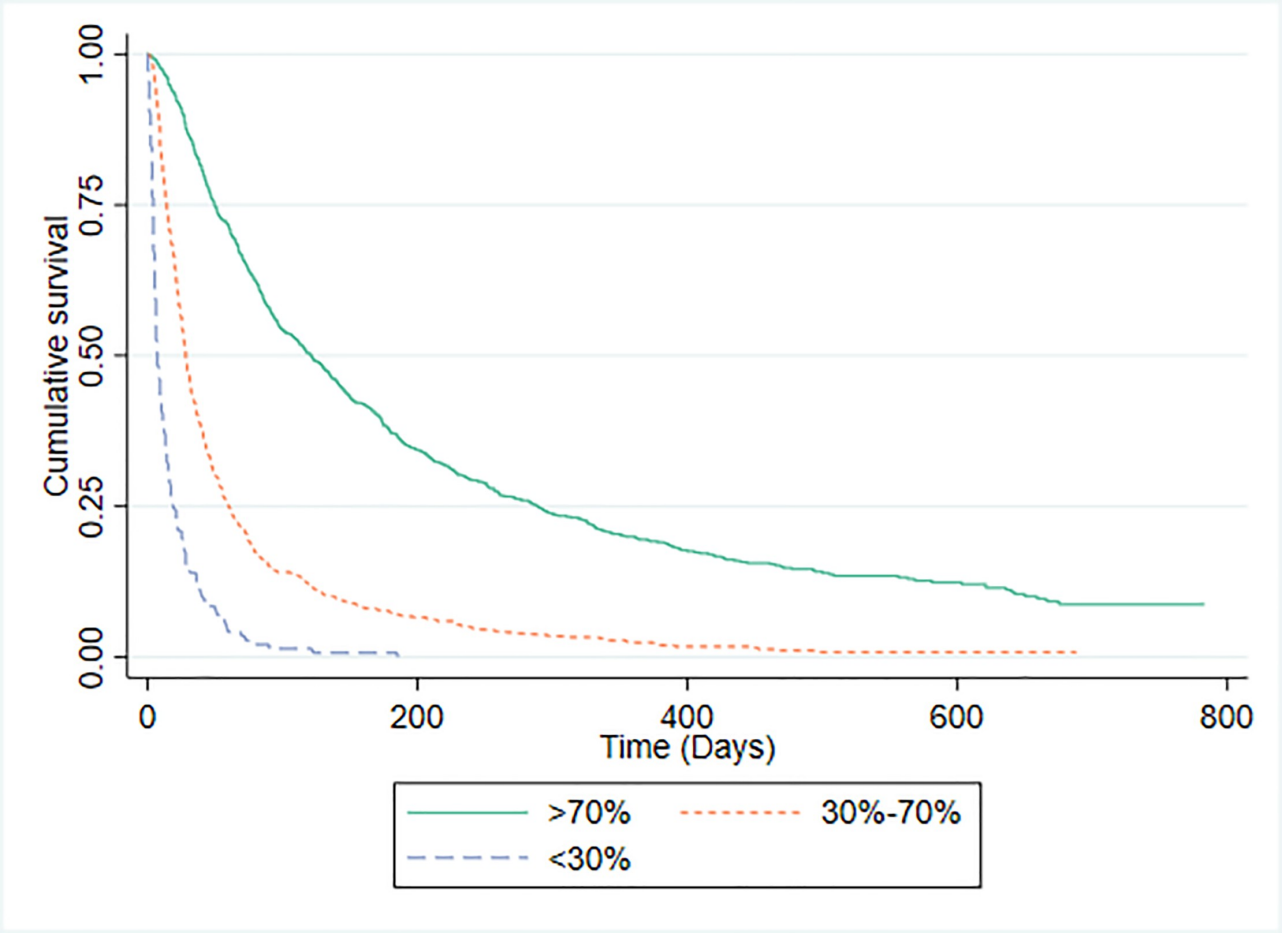
Kaplan-Meier survival curves for PaP risk groups.

**Fig 2:**
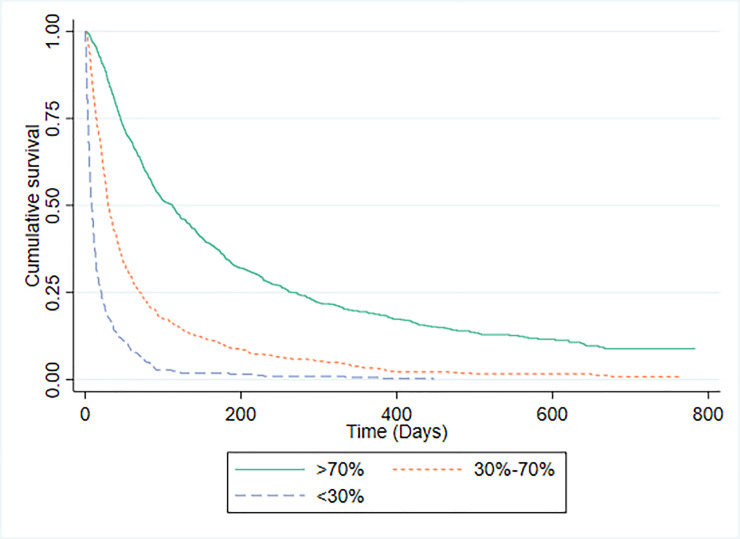
Kaplan-Meier survival curves according to CPS.

Survival data were available for 1592 patients in whom PaP scores were recorded. The C-statistic for the PaP model was 0.771 (95% CI 0.749 to 0.792). There were 794 (49.1%) study participants in risk group A (predicted >70% probability of surviving 30-days); 655 (41.1%) in risk group B (predicted 30% to 70% probability of surviving 30-days); and 143 (9.0%) in risk group C (predicted <30% probability of surviving 30-days). The discrimination of PaP was investigated by plotting survival curves for each risk group, which are shown in Fig 1. The median (IQR) survival for patients for whom PaP scores were available was 51 days (21 to 151). The median (IQR) survival and observed proportion of patients actually surviving 30 days or more for each risk group was: group A 121 days (49 to 289), 86.5% (687/794); group B 28 days (14 to 60), 46.8% (306/655); and group C, 7 days (4 to 19), 15.4% (22/143). For comparison, the 1592 patients were also divided into three groups according to their probability of survival using CPS (Fig 2). The median (IQR) survival and the observed proportion of patients actually surviving 30 days or more for each CPS risk group was: group A 112 days (45 to 272), 85.3% (674/790); group B 30 days (15 to 73), 49.3% (292/593); and group C, 11 days (5 to 27), 23.4% (49/209).

### Feliu Prognostic Nomogram (FPN)

The discriminatory ability of FPN was assessed using Harrell’s C-index. For the FPN model, C-index was 0.684 (95% CI: 0.669 to 0.700; n = 1432) which is somewhat low. The calibration slope for FPN was 1.049 (95% confidence interval: 0.939 to 1.158) which is well-calibrated. The FPN predicted probability of surviving 15 days [median (IQR)] was 72.0% (62.9–81.0) which was similar to the CPS predicted probability of surviving 15 days, which was 80% (70–95). Both predictions were compatible with the actual observed probability of surviving 15 days for these 1432 participants [80.7% (1156/1432)]. Similarly the FPN and CPS predicted probabilities of surviving 30-days were 50.3% (37.9–64.3) and 70% (50–80) respectively; which were in keeping with the actual observed probability of survival which was 65.1% (932/1432). Finally the FPN and CPS predicted probabilities of surviving 60-days were 28.9% (17.3–45.0) and 50% (20–70) respectively; which were similar to the actual observed probability of survival which was 47.1% (675/1432).

### Palliative Performance Scale (PPS)

**Fig 3:**
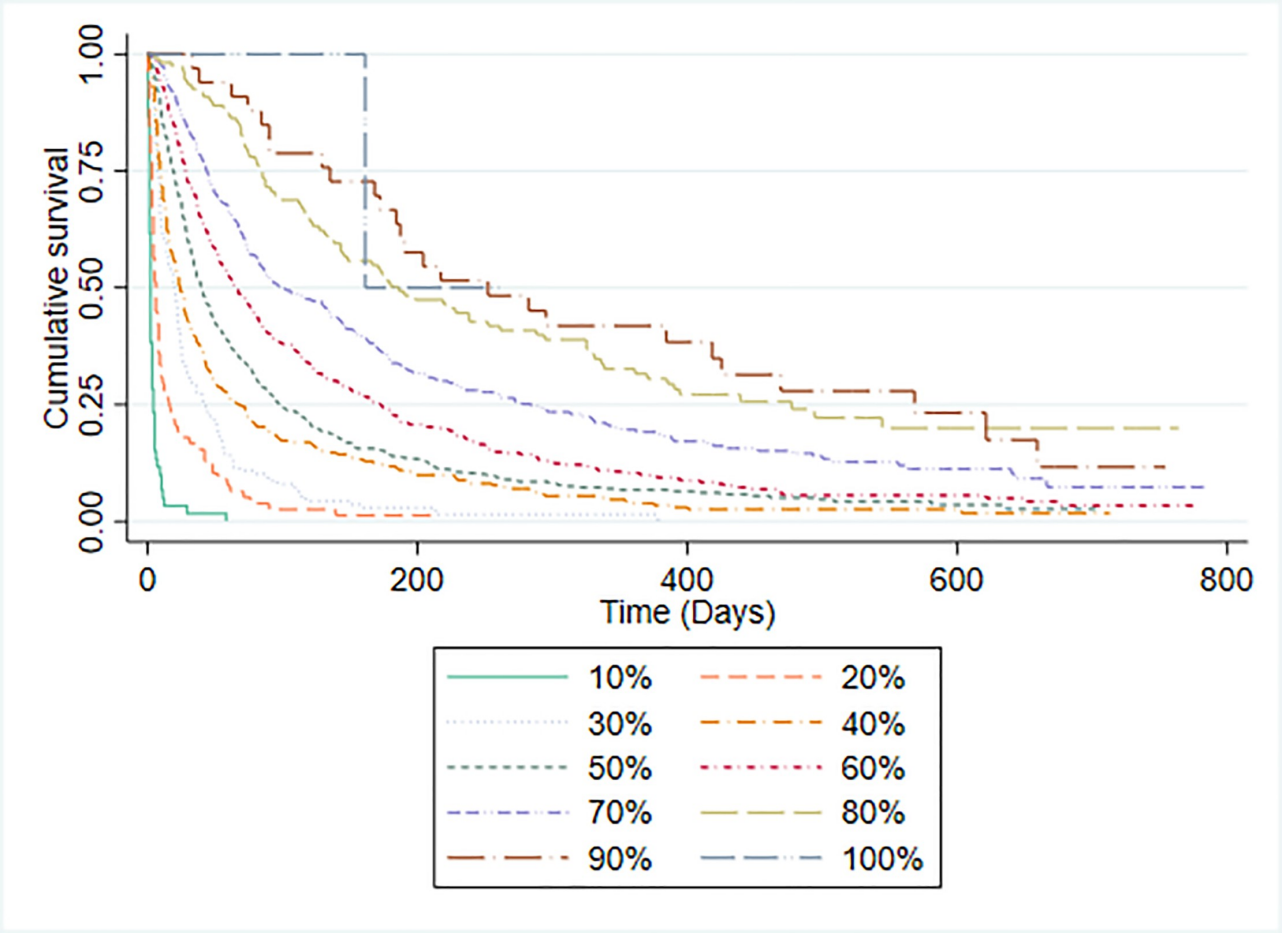
Kaplan-Meier survival curves by PPS level.

**Fig 4:**
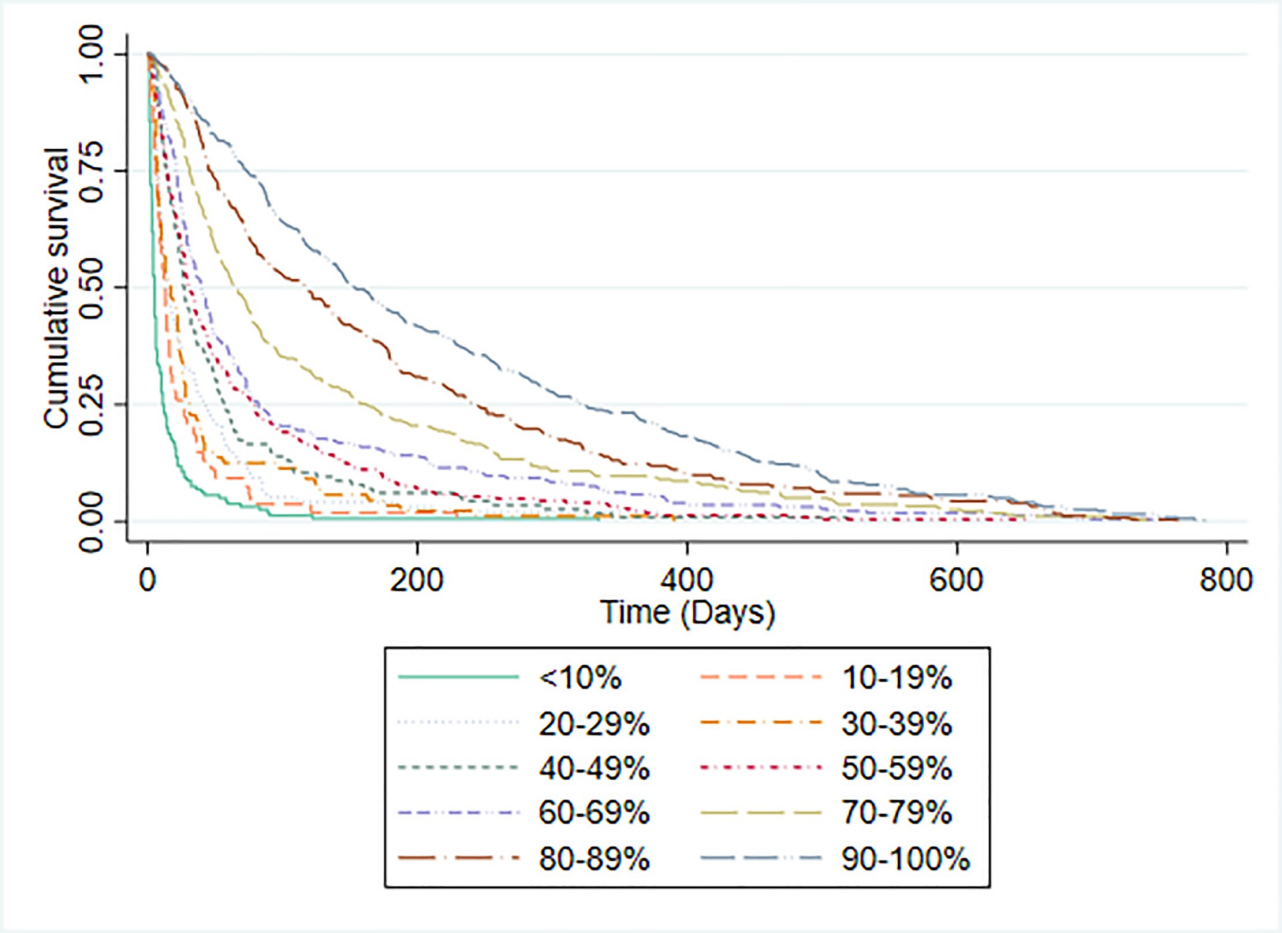
Kaplan-Meier survival curves by 30-day CPS.

PPS scores and median survival time for participants are shown in Table 3. The C-statistic for the PPS was 0.757 (95% CI 0.735 to 0.778). The discriminatory ability of PPS was assessed by plotting Kaplan-Meier survival curves for each PPS level (Fig 3). With the exception of PPS 100% (for which there were too few participants to judge), the median survival of each group increased in the expected direction. PPS was not specifically developed as a prognostic tool, although it has previously been used to categorise patients into prognostic groups [30]. It was therefore not possible to make a straightforward comparison between the accuracy of PPS predictions and CPS. However, we also evaluated the ability of clinicians to categorise patients into ten iso-prognostic groups by plotting the Kaplan-Meier survival curves for patients with different CPS predicted probabilities of surviving 30-days (in 10% increments). Fig 4 illustrates that clinicians were able to stratify patients in a similar way to PPS.

**Table 3:** Number of participants and median survival time in each of the PPS categories.

PPS score	n (%)	Median survival time in days (interquartile range)
10%	60 (3.3)	2 (1 to 4)
20%	78 (4.3)	6 (3 to 16)
30%	138 (7.6)	20 (7 to 42)
40%	273 (14.9)	24 (10 to 69)
50%	493 (27.0)	40 (19 to 97)
60%	376 (20.6)	65 (28 to 172)
70%	265 (14.5)	99 (44 to 284)
80%	109 (6)	186 (85 to 477)
90%	33 (1.8)	252 (135 to 568)
100%	2 (0.1)*	-

*too few participants to judge survival times.

### Palliative Prognostic Index (PPI)

**Fig 5:**
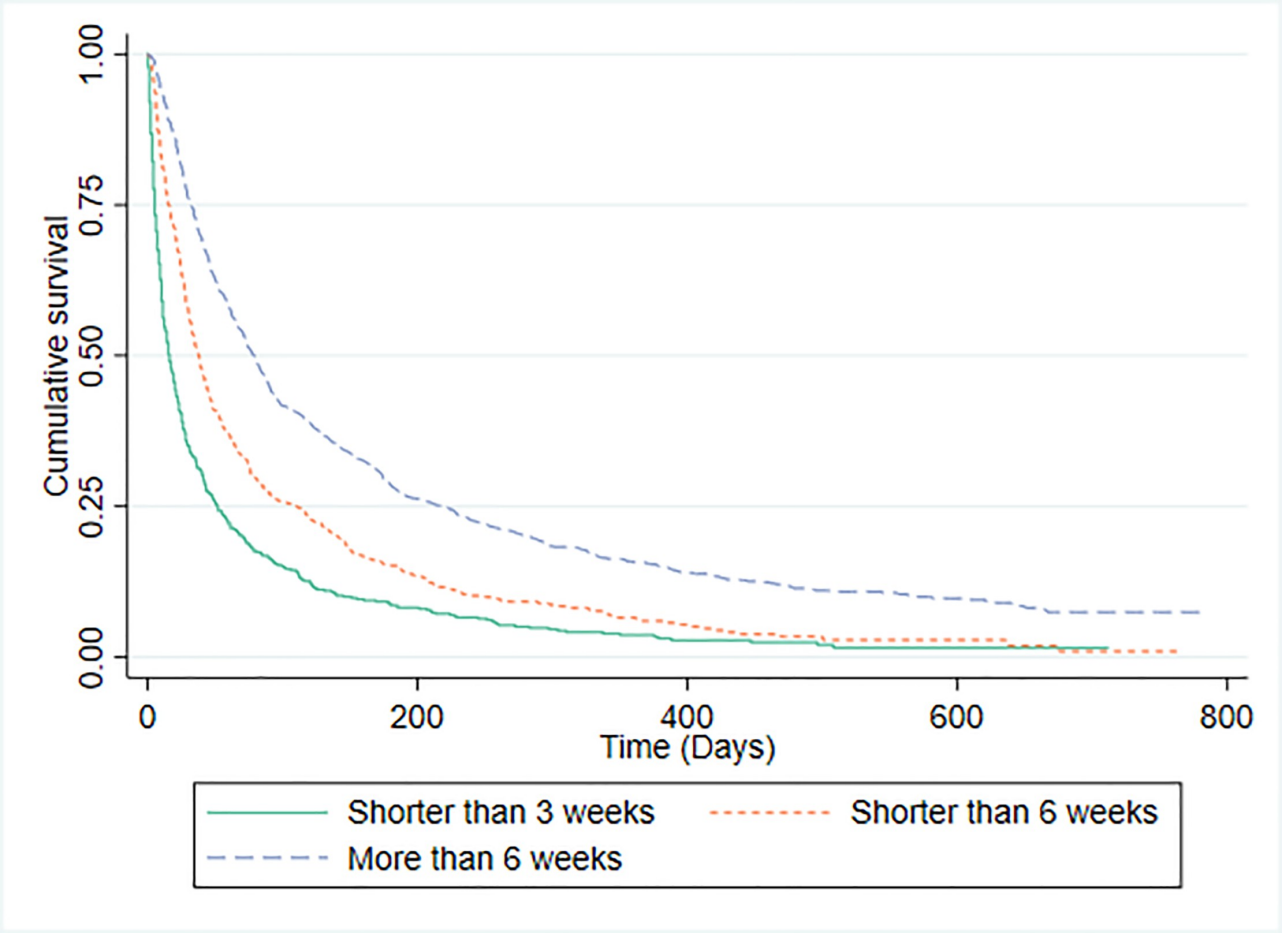
Kaplan-Meier survival curves for PPI predictions.

PPI stratifies patients into three prognostic groups: 501 (49.2%) participants had a PPI score of >6; 428 (23.4%) had a score of >4; and 900 (49.2%) had a score of < = 4. The discrimination of PPI was investigated by plotting Kaplan-Meier survival curves for each risk group (Fig 5) and by calculating C-statistics. For the PPI risk category predicting survival shorter than 3 weeks (n = 1829) C-statistic was 0.675 (0.652 to 0.699) and for PPI risk category predicting survival more than 6 weeks (n = 1829) C-statistic was 0.655 (0.633 to 0.676). The median (IQR) survival of patients in PPI risk groups was: predicted survival shorter than 3 weeks, 16 days (2–52); predicted survival shorter than 6 weeks, 38 days (15–106); and predicted survival of more than 6 weeks, 79 days (32–219). Table 4 provides a comparison of CPS and PPI model predictions compared to actual, observed survival. Overall PPI correctly predicted the outcome on 990/1828 (54.2%) occasions and CPS was correct on 1143/1828 (62.5%) of occasions. The proportion of overall patient deaths predicted correctly by PPI was statistically significantly lower than the corresponding proportion predicted correctly by CPS (p < 0.001).

**Table 4:** Comparison between accuracy of PPI predictions versus accuracy of CPS.

Variable	Doctors’ predictions compared to observed deaths		
PPI predictions compared to observed deaths	CPS was correct	CPS was incorrect	Total
PPI prediction was correct	745 (40.8%)	245 (13.4%)	990
PPI prediction was incorrect	398 (21.8%)	440 (24.1%)	838
Total	1143	685	1828

## Discussion

Previous prognostic studies have validated various prognostic tools statistically and have reported their discrimination, calibration and accuracy [23–25,30–33]. However, the current default method for predicting survival in palliative care practice is to use CPS. Therefore, it is important to use CPS as a benchmark against which to judge performance. We found that clinicians were as capable as PaP at sorting patients into three prognostic groups based on their probability of surviving 30-days. CPS was as capable as FPN at sorting patients into groups according to probability of surviving 15, 30 or 60 days. Although at 30 and 60 days the CPS prediction was closer to actual survival than the FPN prediction, this difference did not reach statistical significance. PPI was significantly less good than CPS at predicting which patients would live for 3 or 6 weeks. Although PPS could not be directly compared against CPS, we found that both approaches were similarly capable of sorting patients into iso-prognostic groups.

There are major methodological challenges to directly comparing the accuracy of prognostic risk models with the accuracy of CPS [16,18,34] and this is one of the limitations with our own analysis. PaP, for example, does not make specific predictions about whether a patient will or will not survive for 30 days, rather it categorises patients into one of three risk groups with different probabilities of surviving for this number of days. We confirmed that PaP scores distinguish between patients with differing probabilities of surviving 30 days (Group A, B and C had an 86.5%, 46.8% and 15.4% probability of surviving 30-days respectively). Superficially, therefore, this would seem to validate use of PaP as a prognostic tool, since the actual survival probabilities of the three groups fall within the margins predicted (i.e. >70%, 30–70% and >70%). However, as our study has demonstrated, clinicians are equally able to distinguish between patients with differing probabilities of surviving 30-days. Using the same risk categories as PaP, clinicians’ sorted patients into groups with survival probabilities of 85.2%, 49.7% and 17.7% respectively.

To simplify the methodological problems, some authors have evaluated PaP as a continuous prognostic variable (ranging between 0–17.5) and have compared it to temporal survival predictions made by clinicians [18,34–36]. Using this approach a higher PaP score is simply regarded as representing a worse prognosis and the three prognostic risk groups as described in the original development study are ignored. [31] Using this method Hui and colleagues [18] reported that the C-index for PaP was 0.64 (0.54 to 0.74) and that this was significantly better (p<0.0001) than the C-index for CPS (0.56 [0.46 to 0.66]). Similarly, Ermacora et al [34] reported that the AUC of PaP was 0.82 (0.77 to 0.86) and this was higher than the AUC reported for two physicians.

Although ignoring the published risk categories, makes comparison of PaP with CPS easier, it is important to consider how the risk categories would actually be interpreted and used in clinical practice. Consider, for example, what it would mean if PaP categorised someone as being in risk group B (with a 30% to 70% probability of surviving 30 days). How should one judge in practice whether, or not, such a prediction was better or worse than a survival prediction made by a clinician? It could be compatible with either outcome (died or survived for 30-days) and clinicians are equally able to identify patients with a 30%-70% chance of surviving 30-days using clinical intuition alone. Moreover, this example illustrates another problem with PaP, which is that the three prognostic categories that it uses, do not necessarily have face validity or clinical utility for clinicians, patients or carers. Indeed, previous studies have reported that many patients prefer not to receive prognostic information in terms of statistical probabilities [37,38]. We have reported elsewhere that most patients, carers and HCPs prefer prognosis being expressed in terms of general categories such as days, weeks or months [21].

FPN was included in this study because, it was one of few prognostic tools to have had its performance evaluated against the performance of other measures [25]. However, since its publication, FPN has not been validated by any independent groups. Since FPN does not make a temporal prediction of survival, it is difficult to directly compare its accuracy to that of clinicians. In the FPN developmental study the C-index was 0.70 and it was well-calibrated. We have confirmed these findings in the current study and moreover we found that FPN was as capable as CPS at sorting patients into groups according to their probability of surviving 15, 30 or 60 days, although CPS was somewhat more accurate at 30 and 60 days. Nonetheless we found that the discriminatory ability of FPN was lower than that of the PiPS-A or PiPS-B models [21].

PPS was not specifically designed to be used as a prognostic tool. However, in a large (n = 6066) retrospective analysis [30] of referrals to a Canadian hospice service, PPS was found to discriminate between groups with different survival prospects. Lau and colleague reported that the median survival of patients with PPS of 10% was 1 day; with PPS 20% was 2 days; with PPS 30% was 5 days; with PPS 40% was 13 days; with PPS 50% was 28 days; with PPS 60% was 43 days and with PPS 70% was 63 days. We similarly found that PPS was able to discriminate between groups with different survival prospects although the median survival for each PPS group was different in our population. Since PPS does not make specific survival predictions it was not possible to directly compare its performance to that of clinicians, although we did find that CPS alone was also able to discriminate patients into multiple iso-prognostic groups.

PPI was developed by Morita and colleagues to predict whether patients would survive for 3 or 6 weeks [22]. Previous studies have reported that PPI has good discrimination, calibration, sensitivity and specificity [17,32,39,40]. Patients with PPI score ≤4 are predicted to survive greater than six weeks. If PPI is >6 then the patient is predicted to survive fewer than three weeks. It was, therefore, relatively straightforward to compare the accuracy of PPI predictions against CPS at three or six weeks. We found that CPS was significantly (p<0.001) better than PPI when performance was directly compared on this metric. However, none of the other published studies have directly compared the accuracy of the PPI and clinicians in this manner [16,17,34,36,41]. Studies have reported on the performance of the PPI for predicting survival at 3 and 6 weeks (which the PPI is designed to predict) [34]; but also its performance at predicting 30-day [17,36]; 100-day [17]; or 90-day survival [16], which it is not.

Our results suggest that practitioners need to exercise caution before incorporating prognostic tools into clinical practice. We did not find evidence that the prognostic tools assessed were more accurate than clinical predictions of survival, nonetheless there are still reasons to believe that their routine use may be a valuable addition to clinical practice. Clinicians may value a prognostic tool even if it were no better than a CPS because it is likely to be be more objective and reproducible and because it could be used as an educational, training or communication aid for less experienced staff. Clinicians find that prognosticating is an uncomfortable task and they are sometimes tempted to avoid discussing time scales, instead choosing to give only vague estimates or avoiding the issue altogether. Since they are more objective and reproducible than clinicians subjective assessments, prognostic tools may also have a role in defining entry criteria to clinical studies or in describing the case mix of clinical services. Future work should focus on evaluating the relative impact of prognostic tools or CPS on clinical care and decision-making and evaluating whether they have other attributes to recommend them (e.g. ease of understanding, reliability or objectivity) beyond their ability to prognosticate accurately.
