# Correction: Prognostic tools or clinical predictions: Which are better in palliative care?

**DOI:** 10.1371/journal.pone.0251757

**Published:** 2021-05-11

**Authors:** The PLOS ONE Staff

## Notice of republication

A file unintended for publication was erroneously included in the originally published article. The publisher apologizes for the error. This article was republished on May 3, 2021, to correct for this error. Please download this article again to view the correct version.
